# Reduced type II interleukin-4 receptor signalling drives initiation, but not progression, of colorectal carcinogenesis: evidence from transgenic mouse models and human case–control epidemiological observations

**DOI:** 10.1093/carcin/bgt222

**Published:** 2013-06-19

**Authors:** Nicola Ingram, Emma L. Northwood, Sarah L. Perry, Gemma Marston, Helen Snowden, John C. Taylor, Nigel Scott, D. Timothy Bishop, P. Louise Coletta, Mark A. Hull

**Affiliations:** Section of Molecular Gastroenterology, Leeds Institute of Biomedical and Clinical Sciences, Wellcome Trust Brenner Building, St James’s University Hospital, Leeds, LS9 7TF, UK,; ^1^Leeds Cancer Research UK Centre, Section of Epidemiology and Biostatistics, Leeds Institute of Cancer Studies and Pathology, St James’s Hospital, Leeds, LS9 7TF, UK and; ^2^Department of Histopathology, St James’s University Hospital, Leeds, LS9 7TF, UK

## Abstract

We investigated the role of interleukin (IL)-4 receptor (IL-4R) signalling during mouse carcinogen-induced colorectal carcinogenesis and in a case–control genetic epidemiological study of *IL-4Rα* single nucleotide polymorphisms (SNPs). Azoxymethane-induced aberrant crypt focus (ACF; 6 weeks) and tumours (32 weeks) were analysed in wild-type (WT) *BALB/c* mice, as well as in *IL-4Rα* ^*−*^^*/−*^, *IL-13* ^*−/−*^ and ‘double-knockout’ (DKO) animals. Colorectal cancer (CRC) cases (1502) and controls (584) were genotyped for six coding *IL-4Rα* SNPs. The association with CRC risk and CRC-specific mortality was analysed by logistic regression. Lack of IL-4Rα expression was associated with increased ACFs [median 8.5 ACFs per mouse (*IL-4Rα* ^*−/−*^) versus 3 (WT); *P *= 0.007], but no difference in the number of colorectal tumours [mean 1.4 per mouse (*IL-4Rα* ^*−/−*^) versus 2 (WT)], which were smaller and demonstrated reduced nuclear/cytoplasmic β-catenin translocation compared with WT tumours. Tumour-bearing *IL-4Rα* ^*−/−*^ mice had fewer CD11b^+^/Gr1^+^ myeloid-derived suppressor splenocytes than WT animals. *IL-13* ^*−/−*^ mice developed a similar number of ACFs to *IL-4Rα* ^*−/−*^ and DKO mice. There was a significant increase in CRC risk associated with the functional SNP Q576R [odds ratio 1.54 (95% confidence interval 0.94–2.54), *P*_trend_ 0.03 for the minor G allele]. There was no effect of IL-4Rα genotype on either CRC-specific or all-cause mortality. These combined pre-clinical and human data together demonstrate that reduced IL-4R signalling has stage-specific effects on colorectal carcinogenesis (increased CRC initiation and risk but reduced tumour progression and no effect on CRC mortality). These results should prompt evaluation of the effect of pharmacological manipulation of IL-4R signalling on future CRC risk and for CRC treatment.

## Introduction

The relatively long natural history of colorectal carcinogenesis in humans, which, in the majority of cases, occurs via the benign colorectal adenoma (or polyp) followed by progression through to invasive colorectal adenocarcinoma (or cancer), makes colorectal cancer (CRC) particularly amenable to prevention strategies (1). Despite significant advances in CRC prevention measures, particularly population screening and surveillance based on early detection of CRC and polyp removal (2,3), there is still a need for alternative CRC prevention strategies such as chemoprevention that could be used to minimize the need for invasive endoscopic investigations and the incidence of interval neoplasia (4).

The relationship between chronic inflammation and different stages of both colitis-associated and ‘sporadic’ colorectal carcinogenesis, from initiation of epithelial cell dysplasia through to promotion of metastasis, is firmly established (5). In a small number of instances, the role of individual pro-inflammatory genes and mediators linking inflammation and intestinal tumorigenesis has been delineated (5). For example, the pro-inflammatory transcription factor nuclear factor κB has been demonstrated to drive murine intestinal tumour initiation and growth, but by different mechanisms involving nuclear factor κB activation in epithelial cells and in stromal inflammatory cells, respectively (6). This nuclear factor κB paradigm highlights that an individual pro-inflammatory signalling pathway may have different roles during initiation, promotion or progression of colorectal carcinogenesis.

There is some experimental evidence that signalling by the T helper 2 cytokines interleukin (IL)-4 and IL-13 also has differential effects depending on the stage of colorectal carcinogenesis that is considered. IL-4 and IL-13 signal via two distinct receptor heterodimers, both of which contain an IL-4 receptor (IL-4R) α chain as a common subunit together with either a cytokine receptor common γ chain (termed the type I IL-4R) or complexed with a IL-13Rα1 chain (termed the type II IL-4R) (7). The type I IL-4R binds IL-4 exclusively and is expressed predominantly by haematopoietic cells. The type II IL-4R can bind both IL-4 and IL-13 and is found mainly in non-haematopoietic cells such as epithelial cells (8). Signal transduction downstream of both type I and type II IL-4Rs occurs via a signal transducer and activator of transcription-6 (STAT-6) pathway, which is initiated by receptor dimerization-induced phosphorylation of the IL-4Rα chain by Janus kinase (8). IL-13 can also bind a separate receptor IL-13Rα2, expression of which is induced by type II IL-4R activation. IL-13Rα2 was previously believed to be a decoy receptor, but IL-13–IL-13Rα2 binding has also been implicated in intestinal fibrogenesis via transforming growth factor β (9).

IL-4Rα chain expression has been demonstrated to be increased in many human cancers including CRC (10,11) and IL-4R signalling has been implicated in driving tumour growth and metastasis in rodent models of advanced CRC (8,12–14). Consistent with these data, tumour cell-derived IL-4 has been demonstrated to induce antiapoptotic protein expression in primary human CRC cells (15). IL-4Rα-mediated signalling also plays a role in the host antitumour immune response mediating immunosuppressive activity of tumour-induced myeloid-derived suppressor cells (MDSCs) (16).

The role of IL-4R signalling at early stages of colorectal carcinogenesis, which are more relevant to CRC prevention, has received less attention than activity in CRC models. In the azoxymethane (AOM) mouse model of ‘sporadic’ CRC initiation, we have previously demonstrated that lack of the *IL-4Rα* gene was associated with an increased number of aberrant crypt focus (ACF), an established biomarker of CRC in rodent chemical carcinogenesis models (17). In contrast with the conclusions from the rodent CRC models, these data suggested a protective, antineoplastic role of IL-4Rα-dependent signalling during AOM-induced initiation of colorectal carcinogenesis in *BALB/c* mice, but stopped short of determining the individual roles of the T helper 2 cytokines and the IL-4Rs in protection from carcinogen-induced carcinogenesis. In addition, two studies have investigated the role of IL-4R signalling in mouse models of colitis-associated CRC (CAC) (11,18). Osawa *et al.* (18) reported that *IL-4*-null *BALB/c* mice developed fewer colorectal tumours (although these authors noted frequent ACFs) than wild-type (WT) animals when treated with AOM alone but noted no difference in tumour incidence between the two genotypes when AOM was used with trinitrobenzene sulphonic acid in order to model CAC. On the other hand, tumour multiplicity was reduced in *IL-4Rα* ‘knockout’ mice compared with WT animals in the AOM/dextran sulphate sodium model of CAC (11).

Given conflicting data on the effect of lack of IL-4Rα-mediated signalling and *IL-4* gene deletion on AOM-induced colorectal carcinogenesis, we sought to investigate the effect of genetic deletion of *IL-4Rα* on tumour development and growth in the AOM model of ‘sporadic’ colorectal carcinogenesis. In addition, we used ‘double-knockout’ (DKO) *IL-4Rα* ^*−/−*^ × *IL-13* ^*−/−*^ mice in order to investigate further the mechanisms underlying the pro-tumorigenic effect of lack of IL-4Rα-mediated signalling.

Functional single nucleotide polymorphisms (SNPs) are recognized in the human IL-4Rα gene with SNP rs1801275 being best characterized as a guanine for adenine substitution at nucleotide 1902 (1902A>G), leading to a change from a glutamine to arginine residue at position 576 (Q576R) adjacent to a tyrosine phosphorylation site (Y575) in the cytoplasmic domain of IL-4Rα-chain (19,20). Early studies suggested that Q576R was associated with reduced SHP-1 phosphotyrosine phosphatase binding to the IL-4Rα chain and presumed loss of phosphatase activity against substrates including STAT-6 (19). However, subsequent studies in human and mouse leucocytes did not find evidence of increased STAT-6 phosphorylation or downstream DNA binding (20,21). Moreover, the Q576R substitution and the IL-4Rα SNP rs1805015 (S503P) have also been associated with reduced phosphorylation of STAT-6, as well as increased phosphorylation of insulin receptor-like substrate (IRS) 1 and 2 in human peripheral blood mononuclear cells consistent with Q576R as a ‘loss of function’ SNP (22). Importantly, similar studies have not yet been performed in non-haematopoietic cells expressing the type II IL-4R. Therefore, in parallel with our mechanistic mouse studies, we tested the hypothesis that ‘functional’ IL-4Rα SNPs, including the putative ‘loss of function’ SNP Q576R, are associated with increased CRC risk in a case–control human epidemiological study.

## Methods

### Animal studies

About 6- to 8-week-old male and female *BALB/c* animals from our existing colony of WT and IL-4Rα^−/−^ mice were used (17). *BALB/c* mice harbouring a homozygous deletion of the IL-13 gene (IL-13^−/−^) were a kind gift from Prof. Andrew McKenzie, University of Cambridge (23). DKO IL-4Rα^−/−^ × IL*-13* ^*−/−*^ mice were bred and genotyped by PCR (see Supplementary Methods, available at *Carcinogenesis *Online).

Animals received either two intraperitoneal injections of 10mg/kg AOM (Sigma) or saline (sham) 1 week apart followed by killing 6 weeks later (ACF protocol), or received weekly 10mg/kg AOM injections or sham saline injections for 6 weeks, for analysis 32 weeks later (tumour protocol).

### Tissue/blood collection and analysis

In the tumour protocol, mice were administered 250 μl of 5-bromo-deoxyuridine (BrdU; ~0.75mg/kg BrdU, Cell Proliferation Labelling Reagent; GE Healthcare, Little Chalfont, UK) intraperitoneally 2 h before killing. All animals were killed by CO_2_ asphyxiation. Whole blood was obtained immediately after death by cardiac puncture.

Blood collection and haemocytometer analysis are described in Supplementary Methods, available at *Carcinogenesis *Online.

The whole colon was removed and flushed with phosphate-buffered saline. For the ACF protocol, the colon was opened, cleaned and fixed in 4% (wt/vol) paraformaldehyde in phosphate-buffered saline as described (17). For the tumour protocol at 32 weeks, the colon was first opened longitudinally onto a glass plate for examination of macroscopic tumours using an Olympus SZ60 stereo-microscope fitted with a graticule (Southend, UK). The presence, position and maximum diameter of each tumour were noted before excision for fixation in 4% paraformaldehyde overnight. The remaining colon was fixed separately in 4% paraformaldehyde for ACF counting. Fixed tissue was washed and stored in 70% (vol/vol) ethanol before embedding in paraffin.

The number and size (number of crypts per ACF) of ACFs were measured in methylene blue-stained fixed colon as described (17).

Splenocytes were obtained by mashing the whole spleen through a 70 μm mesh filter for immediate storage at −80°C in 90% (vol/vol) fetal calf serum and 10% (vol/vol) dimethylsulphoxide.

### Flow cytometry and cytokine analysis

One million splenocytes were incubated with antibodies or an isotype control for 30 min at 4°C. Cells were analysed on a BD LSRII fluorescence-activated cell sorting analyser using fluorescence-activated cell sorting Diva software. Cytokine levels were measured by multiplex immunoassay. For more information on flow cytometry and cytokine analysis, see Supplementary Methods, available at *Carcinogenesis *Online).

### Immunohistochemistry

See Supplementary Methods, available at *Carcinogenesis *Online.

### Statistical analysis

GraphPad Prism 5.02 software (GraphPad Software, San Diego, CA) was used to analyse the data from the experimental studies. *P* ≤ 0.05 was considered to be statistically significant.

### The case–control epidemiology study

Patients with CRC were recruited between 1992 and 2011 from NHS hospitals in Leeds and Harrogate as reported previously (24,25). Patients were invited to participate after a confirmed diagnosis of adenocarcinoma of the colorectum. Only cases that were recruited within 2 years of the date of CRC diagnosis were included. Cases were observed until death or the censor date of 20 June 2011. In the all-cause mortality survival analysis, death from any cause was the end point, whereas in the CRC-specific survival analysis, death from CRC was the end point and death from other causes was censored. Healthy, population-based, age-matched controls with no history of cancer were recruited via the same General Practice surgery or cases (spouses or friends) (24,25).

Six SNPs in the coding region of the human *IL-4Rα* gene [rs1801275 (Q576R), rs1805015 (S503P), rs1805010 (I75V), rs1805011 (A400E), rs1805013 (S436L) and rs1805016 (A752S)] were genotyped using Taqman-based allelic discrimination analysis. About 6% of DNA samples were randomly duplicated across the different well plates for validation.

Detailed methodology is described in Supplementary Methods, available at *Carcinogenesis *Online. The distribution of age at diagnosis, sex, body mass index (BMI) at interview, smoking status (ever/never), taking vigorous physical activity and continuous non-steroidal anti-inflammatory drug (NSAID) use were compared between cases and controls. Odds ratios (ORs) and 95% confidence intervals (CIs) were calculated. The genotype distribution for each SNP was tested in controls for deviation from Hardy–Weinberg equilibrium. Genotype frequencies were compared between cases and controls using Pearson’s chi square test. ORs and 95% CIs were calculated from unconditional logistic regression, first unadjusted and then adjusted for sex and NSAID use, treating homozygosity for the common reference allele as the baseline risk category. For each SNP, crude hazard ratios and 95% CIs were estimated from Cox proportional hazards models for both CRC-specific mortality and all-cause mortality (see Supplementary Methods, available at *Carcinogenesis *Online, for more detail).

For a polymorphism with a risk genotype frequency of 12% in the control population, this study had >80% power to detect an OR of ≥1.5 at a significance level of 0.05. The epidemiological data were analysed using Intercooled STATA version 9.0 (StataCorp LP, College Station, TX).

## Results

### Lack of IL-4Rα-mediated signalling is associated with increased AOM-induced ACF multiplicity but does not drive colorectal tumour development or growth

**Fig. 1.**
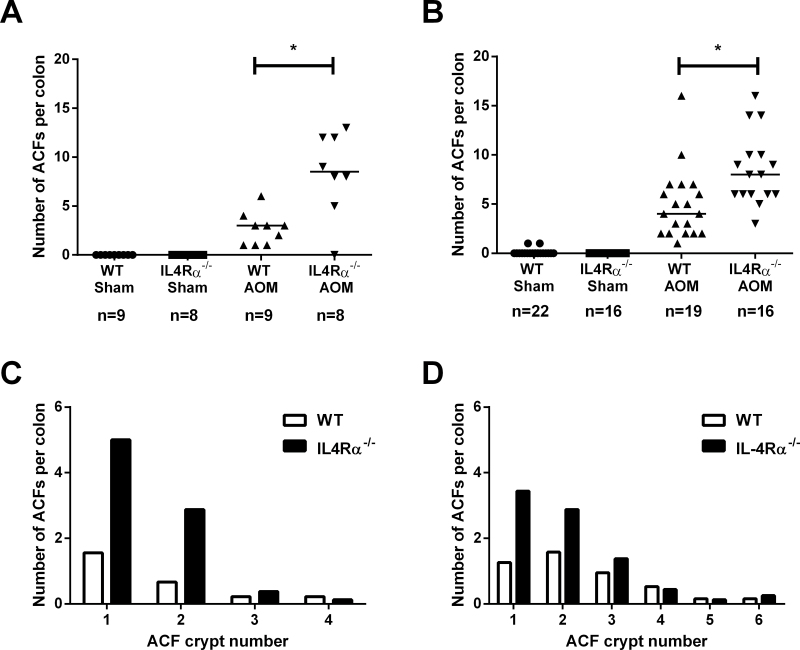
Lack of *IL-4Ra*-mediated signalling is associated with an increase in AOM-induced ACF multiplicity. (**A**) The number of ACFs per colon 6 weeks after sham or AOM treatment in WT and *IL-4Rα* ^*−/−*^ mice. Data are from individual mice. Bars represent the median value for each group. The number of mice within each group is noted below the treatment group labels. *P = 0.007; Mann–Whitney *U*-test. (**B**) The number of ACFs per colon 32 weeks after sham or AOM treatment in WT and *IL-4Rα* ^*−/−*^ mice. Data are from individual mice. Bars represent the median value for each group. The number of mice within each group is noted below the treatment group labels. *P = 0.002; Mann–Whitney *U*-test. (**C**) ACF size (measured as the number of abnormal crypts per ACF) in WT and *IL-4Rα* ^*−/−*^ mice 6 weeks after AOM administration. (**D**) ACF size in WT and *IL-4Rα* ^*−/−*^ mice 32 weeks after AOM administration. ACF size was not significantly different between WT and *IL-4Rα* ^*−/−*^ mice at either 6 or 32 weeks after AOM administration (both *P* = 0.7; Mann–Whitney *U*-test).

First, in an independent experiment using the ACF protocol, we confirmed our previous finding (17) that the number of colorectal ACFs that developed 6 weeks after AOM administration in *BALB/c IL-4Rα* ^*−/−*^ mice [median 8.5 ACFs per mouse (interquartile range, IQR 5.75–12), *n* = 8] was significantly higher than the ACF multiplicity in WT controls [median 3 ACFs per mouse (IQR 1–3.5), *n* = 9; *P* = 0.007; Figure 1A]. However, lack of IL-4Rα-mediated signalling was not associated with ACF development in the absence of AOM treatment (Figure 1A).

We also examined a cohort of mice, which had received a prolonged course of AOM, at 32 weeks as per the tumour protocol. At this time point, *IL-4Rα* ^*−/−*^ mice had a median of 8 ACFs per mouse (IQR 6–10; *n* = 16) compared with a median value of 3 ACFs per mouse (IQR 2–7; *n* = 19) in WT animals (*P* = 0.002; Figure 1B). The size of ACFs, measured as the number of abnormal crypts per ACF, did not differ significantly between the two genotypes at either 6 or 32 weeks after AOM administration (Figure 1C and D). There was no difference in ACF multiplicity or size between male and female *IL-4Rα* ^*−/−*^ mice at either 6 weeks [median 9 (IQR 6.5–12.5) ACFs per female *IL-4Rα* ^*−/−*^ mouse (*n* = 5) compared with male animals with a median 8 (IQR 0–12) ACFs (*n* = 3)] or 32 weeks [median 7.5 (IQR 5.75–14) ACFs per female *IL-4Rα* ^*−/−*^ mouse (*n* = 10) compared with male animals median 8 (IQR 6–9.25) ACFs (*n* = 6)].

**Fig. 2.**
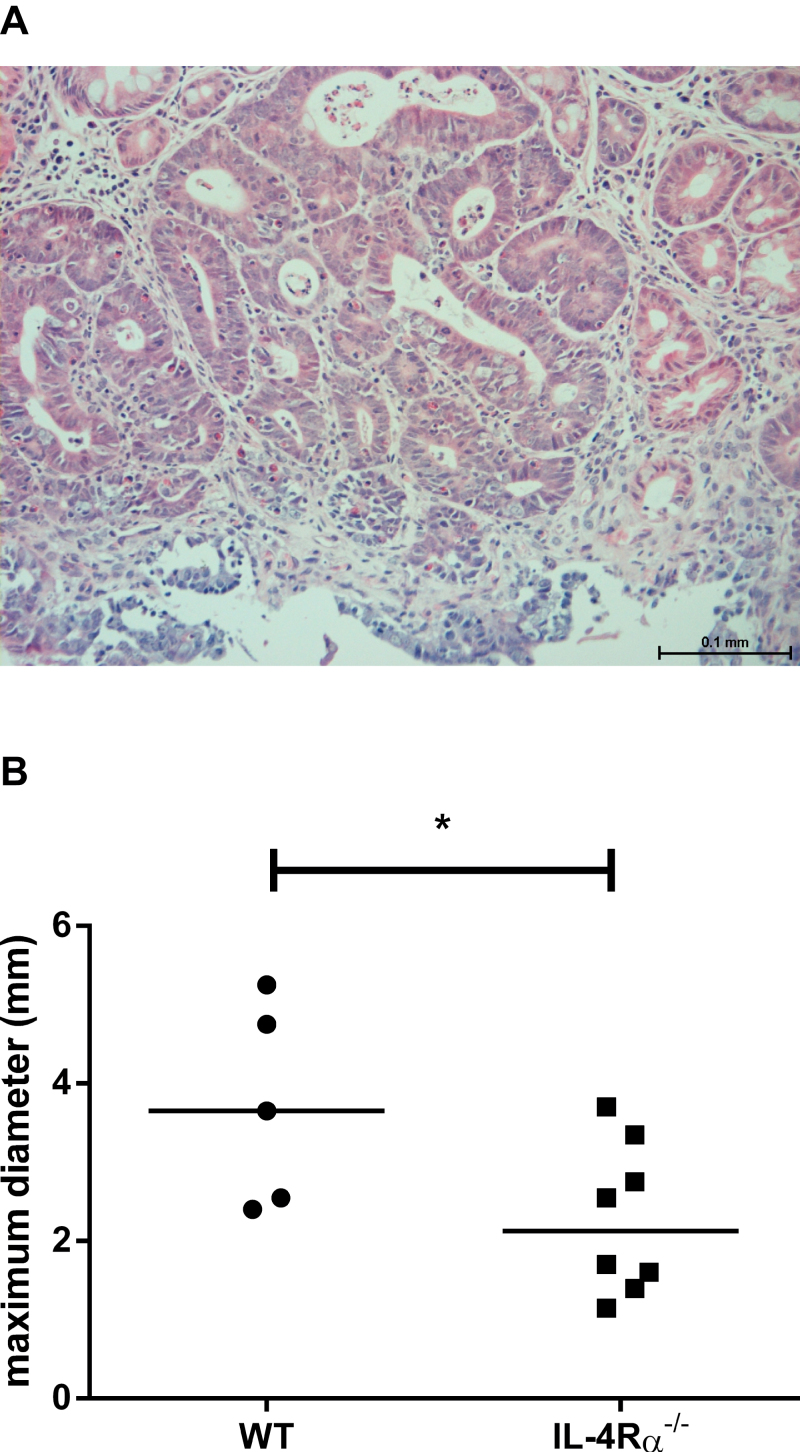
AOM-induced tumour characteristics in *BALB/c* mice. (**A**) All tumours exhibited high-grade dysplasia. There was no invasion of the muscularis mucosa, so tumours were classified as carcinoma *in situ*. Size bar = 100 μm. (**B**) The size (maximum diameter) of colonic tumours in WT and *IL-4Rα* ^*−/−*^ mice. Data points represent individual adenomas and the bars indicate the median value for each group. *P = 0.05 (Mann–Whitney *U*-test) for the difference between genotypes.

No tumours were found in sham-treated animals at 32 weeks. Tumours in AOM-treated mice were restricted to females and were localized exclusively to the distal colon. All tumours were classified as carcinoma-*in* *situ* or high-grade dysplastic adenoma by a consultant histopathologist (Figure 2A). In contrast with the effect of *IL-4Rα* deletion on ACF multiplicity, lack of IL-4Rα-mediated signalling did not promote macroscopic tumour development in female mice. At least 1 tumour was present in 5 of 14 (26.3%) of WT mice (mean 2 tumours per tumour-bearing mouse) compared with 4 of 16 (25%) of *IL-4Rα* ^*−/−*^ mice (mean 1.4 tumours per tumour-bearing animal). However, tumours in *IL-4Rα* ^*−/−*^ mice were significantly smaller than tumours from WT mice [mean (standard deviation, SD) maximum tumour diameter 2.3 (1.0) mm in *IL-4Rα* ^*−/−*^ mice versus 3.7 (1.3) mm in WT mice; *P* = 0.05; Mann–Whitney *U*-test; Figure 2B].

**Fig. 3.**
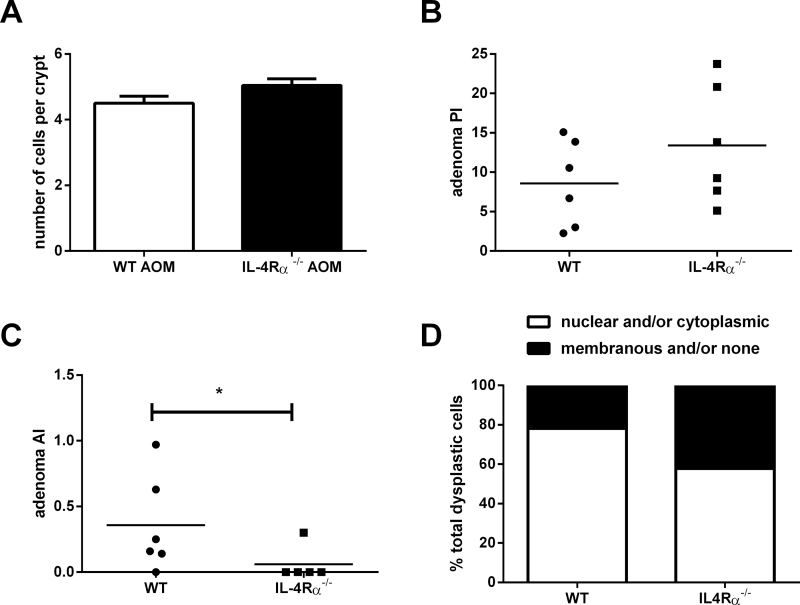
Biomarkers of proliferation, apoptosis and tumour growth at 32 weeks after AOM administration. (**A**) The BrdU crypt proliferation index in non-neoplastic mucosa from WT and *IL-4Rα* ^*−/−*^ mice. For each genotype, data are the mean and standard error of a minimum of 149 crypts counted in 8 separate mice. *P* = 0.25 for the difference between WT and *IL-4Rα* ^*−/−*^ animals (Mann–Whitney *U*-test). (**B**) The BrdU proliferation index in WT (*n* = 6) and *IL-4Rα* ^*−/−*^ (*n* = 6) tumours. Individual tumour proliferation index values are shown with bars representing the mean value. A mean of 1296 (SD = 514) dysplastic epithelial cells per adenoma was counted. *P* = 0.12 for the difference between WT and *IL-4Rα* ^*−/−*^ animals (Mann–Whitney *U*-test). (**C**) Cleaved caspase 3 AI in WT (*n* = 6) and *IL-4Rα* ^*−/−*^ (*n* = 5) tumours. Individual tumour AI values are shown with bars representing the mean value. A mean of 2643 (SD = 582) dysplastic epithelial cells per adenoma was counted (*P = 0.05; Mann–Whitney *U*-test). (**D**) β-Catenin localization in dysplastic cells in AOM-induced colonic tumours from WT and *IL-4Rα* ^*−/−*^ mice. A mean of 860 cells per adenoma was assessed for β-catenin localization by immunohistochemistry. The results are presented as the mean of the percentage of cells with no or membranous β-catenin staining and nuclear and/or cytoplasmic β-catenin staining per adenoma from WT (*n* = 6) and *IL-4Rα* ^*−/−*^ mice (*n* = 6).

There was no significant difference in BrdU positivity of epithelial cells in *IL-4Rα* ^*−/−*^ versus WT mice in either non-neoplastic mucosa or in adenomas (Figure 3A and B) suggesting that differences in epithelial cell proliferation did not explain the differences observed in ACF formation (a biomarker of tumour initiation) or tumour growth. Counter-intuitively, we also observed a significantly lower apoptosis index (AI) in *IL-4Rα* ^*−/−*^ tumours compared with WT tumours (*P* = 0.05; Mann–Whitney *U*-test; Figure 3C). However, smaller *IL-4Rα* ^*−/−*^ tumours contained a higher percentage of cells, which were negative for β-catenin immunoreactivity, or exhibited membranous staining only compared with WT animals (*P* = 0.04, Student’s *t*-test; Figure 3D and Supplementary Figure 1, available at *Carcinogenesis *Online).

### Mechanisms driving intestinal tumorigenesis in the absence of IL-4Rα-mediated signalling

In our previous study of AOM-induced intestinal tumorigenesis using a short-term ACF protocol, we demonstrated that AOM administration was associated with a dramatic increase in serum tumour necrosis factor α level 6 weeks post-AOM injection and that genetic deletion of the IL-4Rα chain was associated with raised serum IL-4 levels (16). When we repeated this analysis at 32 weeks using the tumour AOM protocol, we observed that there was now no difference in the serum levels of the T helper 1 and 2 cytokines between the two mouse genotypes and that the tumour necrosis factor α response to AOM administration had disappeared, suggesting that both were short-term, reversible phenomena (Supplementary Figure 2, available at *Carcinogenesis *Online). There was also no difference between serum cytokine levels of IL-1β, IL-6, IL-10 and IL-15 between the treatment groups and mouse genotypes (data not shown).

At 32 weeks, there was no significant difference in the number of circulating blood monocytes, granulocytes or lymphocytes between WT and *IL-4Rα* ^*−/−*^ mice (Supplementary Figure 3A, available at *Carcinogenesis *Online). We also examined splenic T lymphocyte (CD4^+^, CD8^+^ and CD25^+^ FoxP3^+^) populations, as well as the number of CD11b^+^ Gr1^+^ MDSCs (Supplementary Figure 3B, available at *Carcinogenesis *Online). We observed that splenic MDSC induction in AOM-induced tumour-bearing animals was absent in *IL-4Rα* ^*−/−*^ mice (Supplementary Figure 3B, available at *Carcinogenesis *Online).

**Fig. 4.**
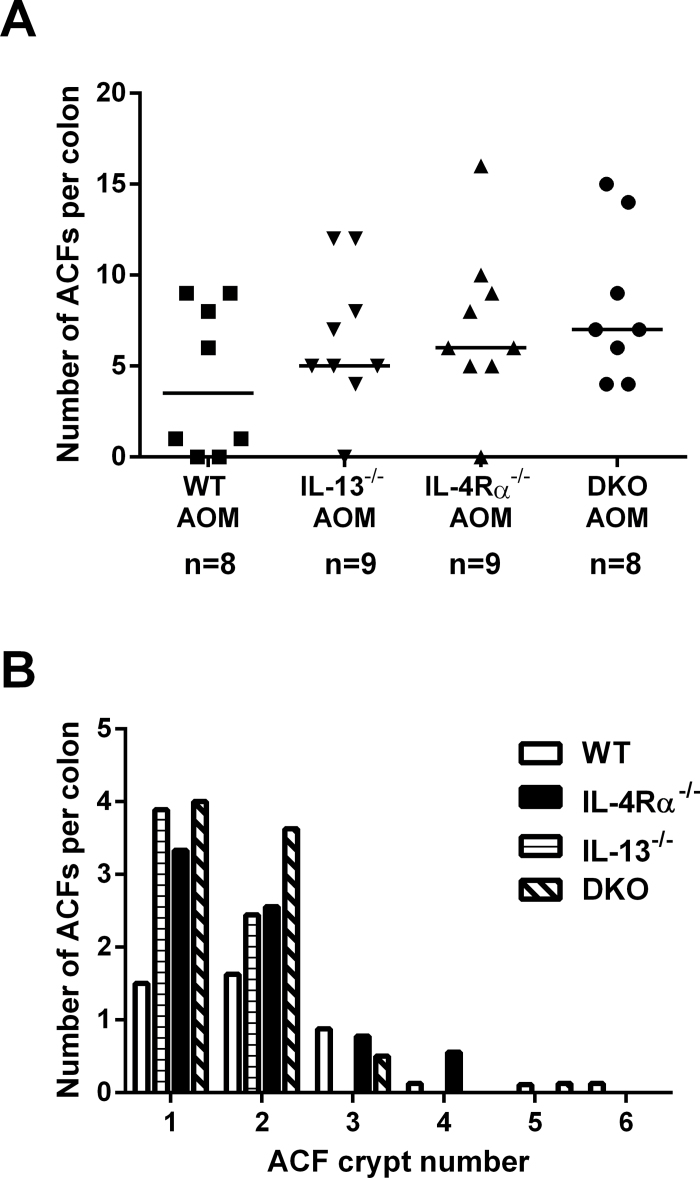
Lack of *IL-13*-mediated signalling does not abrogate the increase in AOM-mediated ACF development in IL-4Rα^−/−^ mice compared with WT animals. (**A**) The number of ACFs per colon 6 weeks after sham or AOM treatment in WT, *IL-13* ^*−/−*^, *IL-4Rα* ^*−/−*^ and *IL-4Rα* ^*−/−*^ *×* *IL-13* ^*−/−*^ DKO mice. Data are from individual mice. Bars represent the median value for each group. The number of mice within each group is noted below the treatment group labels. (**B**) ACF size (measured as the number of abnormal crypts in each ACF) in WT, *IL-13* ^*−/−*^, *IL-4Rα* ^*−/−*^ and DKO mice 6 weeks after AOM administration. There was no significant difference between the genotypes (Kruskal–Wallis test followed by Dunn’s multiple comparison test).

We previously speculated that IL-13Rα2-mediated signalling by IL-13 might account for increased AOM-induced tumour initiation in *IL-4Rα* ^*−/−*^ mice based on increased colorectal mucosal expression of transforming growth factor β, upregulation of which is recognized to occur downstream of IL-13–IL-13Rα2 activation (17). In order to directly test the hypothesis that IL-4Rα-independent signalling by IL-13 contributes to increased initiation of chemical colon carcinogenesis, we compared AOM-induced ACF multiplicity at 6 weeks in *IL-13*-null mice, as well as *IL-13* ^*−/−*^ × *IL-4Rα* ^*−/−*^ DKO animals, with *IL-4Rα* ^*−/−*^ and WT mice. Sham-treated *IL-13* ^*−/−*^ mice had a total of two unicryptal ACFs in five mice (data not shown). *IL-13* ^*−/−*^ animals developed a similar number and size of AOM-induced colorectal ACFs to *IL4Rα* ^*−/−*^ mice, both in excess of WT animals (Figure 4A and B). Lack of IL-13 did not abrogate the effect of absence of IL-4Rα-mediated signalling in DKO animals, thereby refuting the hypothesis that increased tumour initiation in *IL-4Rα* ^*−/−*^ mice is dependent on IL-13 signalling. Instead, this evidence implicates a protective role for the type II IL-4R, for which both IL-4 and IL-13 are ligands, and which is the predominant IL-4R expressed by intestinal epithelial cells (11).

### Functional IL-4Rα SNPs are associated with an increased risk of CRC but no difference in CRC survival

As the experimental studies demonstrated that genetic deletion of *IL-4Rα* is associated with increased tumour initiation, but not accelerated tumour growth, we tested whether altered IL-4Rα-mediated signalling would be associated with increased CRC risk, but a differential effect on CRC outcomes after diagnosis, in a case–control epidemiological study.

We studied 1502 CRC cases and 584 controls, representing >80% of all eligible study participants (Table I) (24,25). About 99% of the total population was white British in origin, with 0.6% Afro-Carribean, 0.6% Asian and 0.1% mixed race. As expected, there was a significantly smaller proportion of females in the CRC cases compared with controls (*P* ≤ 0.001) and significantly fewer CRC cases were continuous NSAID users compared with controls (*P* = 0.008; Table I). Therefore, the logistic regression models to assess the effect of the SNPs on CRC susceptibility were adjusted for sex and NSAID use. A higher proportion of cases than controls had ever smoked, though the difference did not quite reach statistical significance (*P* = 0.06). There were no differences in the distributions of age at diagnosis, BMI at interview or physical activity between cases and controls.

**Table I.** Characteristics of CRC cases and controls in the *IL-4Rα* SNP study

Characteristic	Statistic	**Controls**	**Cases**	**Unadjusted OR (95% CI)**	***P* value** ^**a**^
		**584** ^**b**^	**1502** ^**b**^		
Age (years)	Mean (SD)	67.3 (9.3)	67.3 (10.5)	1.00 (0.99, 1.01)	0.96
Sex					
Male	*n* (%)	273 (46.8)	881 (58.7)	1.0	
Female		311 (53.3)	621 (41.3)	0.62 (0.51, 0.75)	<0.001
Ever smoked					
No	*n* (%)	248 (42.7)	504 (38.2)	1.0	
Yes		333 (57.3)	816 (61.8)	1.21 (0.99, 1.47)	0.06
BMI (kg/m^**2**^)	Mean (SD)	26.3 (4.5)	26.0 (4.3)	0.99 (0.96, 1.01)	0.19
Vigorous physical activity^**c**^					
No	*n* (%)	415 (72.4)	920 (70.3)	1.0	
Yes		158 (27.6)	389 (29.7)	1.11 (0.89, 1.38)	0.35
**NSAID (continuous use)** ^**d**^					
No	*n* (%)	375 (64.5)	932 (70.7)	1.0	
Yes		206 (35.5)	386 (29.3)	0.75 (0.61, 0.93)	0.008

^a^Significance values (*P*) were calculated by Pearson’s chi square for sex, ever smoked and vigorous physical activity. Student’s *t*-test was used for age and BMI comparisons.

^b^The number of cases and controls analysed for each factor varied slightly because of missing data.

^c^Vigorous physical exercise was defined by the question “In a typical week one year ago, did you exercise (includes housework, DIY, gardening, walking) vigorously enough to cause sweating or a faster heartbeat?”

^d^Continuous NSAID use was defined by the question “Have you ever taken NSAIDs on a regular basis for periods of 3 months or longer?”

All genotype frequency distributions in the control group were in accordance with Hardy–Weinberg equilibrium. Haplotype analysis indicated that SNPs rs1805011, rs1805013, rs1805015, rs1805016 and rs1801275 were all in strong linkage disequilibrium (data not shown). The associations between individual SNPs and CRC susceptibility are shown in Table II. The strongest association with CRC susceptibility was observed for SNP rs1801275 (Q576R). Increased risk of CRC was observed in homozygotes of the rare allele (GG) compared with common reference allele homozygotes (AA) with a *P*_trend_ of 0.04 (Table II). In the adjusted analysis, the OR was 1.54 (95% CI 0.94–2.54; *P* = 0.089) for the homozygous G allele. *P*_trend_ for the association between the G allele and CRC risk was 0.03. There were no statistically significant differences in CRC risk in the overall population for the other SNPs investigated (Table II).

**Table II.** The relationship between IL-4Rα SNPs and CRC susceptibility

**SNP**		**Controls**	**Cases**	**Unadjusted**			**Adjusted** ^**a**^		
		***n* (%)** ^**b**^	***n* (%)** ^**b**^	**OR**	**95% CI**	***P* value**	**OR**	**95% CI**	***P* value**
rs1801275	AA	364 (63.1)	847 (58.5)	1.0			1.0		
Q576R	AG	190 (32.9)	524 (36.2)	1.19	(0.96–1.46)	0.107	1.18	(0.96–1.46)	0.121
	GG	23 (4.0)	77 (5.3)	1.44	(0.89–2.33)	0.139	1.54	(0.94–2.54)	0.089
	Trend			1.19	(1.01–1.41)	0.041	1.21	(1.02–1.43)	0.033
rs1805015	TT	400 (69.4)	951 (65.7)	1.0			1.0		
S503P	TC	158 (27.4)	442 (30.5)	1.18	(0.95–1.46)	0.140	1.16	(0.93–1.45)	0.186
	CC	18 (3.1)	54 (3.7)	1.26	(0.73–2.18)	0.404	1.40	(0.79–2.47)	0.249
	Trend			1.16	(0.97–1.38)	0.111	1.17	(0.97–1.40)	0.097
rs1805016^**c**^	TT	525 (91.1)	1288 (89.0)	1.0			1.0		
A752S	TG	51 (8.9)	155 (10.7)	1.24	(0.89–1.73)	0.207	1.27	(0.90–1.79)	0.168
	GG	0 (0.0)	4 (0.3)	NA			NA		
	Trend			1.29	(0.94–1.79)	0.120	1.34	(0.96–1.87)	0.086
rs1805013^**c**^	CC	536 (92.3)	1308 (90.5)	1.0			1.0		
S436L	CT	44 (7.6)	136 (9.4)	1.27	(0.89–1.81)	0.191	1.34	(0.93–1.93)	0.116
	TT	1 (0.2)	2 (0.1)	0.82	(0.07–9.06)	0.871	1.04	(0.09–11.82)	0.976
	Trend			1.24	(0.88–1.74)	0.222	1.31	(0.92–1.87)	0.129
rs1805011	AA	439 (77.3)	1073 (76.0)	1.0			1.0		
A400E	AC	117 (20.6)	305 (21.6)	1.07	(0.84–1.36)	0.599	1.06	(0.83–1.36)	0.628
	CC	12 (2.1)	33 (2.3)	1.13	(0.58–2.20)	0.730	1.14	(0.57–2.24)	0.714
	Trend			1.06	(0.87–1.30)	0.544	1.06	(0.86–1.31)	0.558
rs1805010	AA	162 (28.8)	428 (30.4)	1.0			1.0		
I75V	AG	295 (52.4)	700 (49.7)	0.90	(0.72–1.13)	0.352	0.91	(0.72–1.15)	0.421
	GG	106 (18.8)	280 (19.9)	1.00	(0.75–1.33)	0.999	0.99	(0.73–1.33)	0.928
	Trend			0.99	(0.86–1.14)	0.871	0.98	(0.85–1.14)	0.826

^a^Adjusted for sex and NSAID use.

^b^The total number of cases and controls differ across SNPs reflecting a small number of failed SNP assays.

^c^SNPs rs1805016 and rs1805013 are in strong linkage disequilibrium with a Dʹ value of 0.9.

When stratified by sex, genotype effects on increased CRC susceptibility were stronger than those observed in the overall analysis, with significant increased risks seen only in females for SNPs rs1801275 (Q576R), rs1805015 (S503P) and rs1805011 (A400E; Supplementary Table I, available at *Carcinogenesis *Online). Further assessment of gender and IL-4Rα genotypes in the control population indicated that an increased frequency of the common homozygote for the SNPs in females was driving this statistical interaction. No significant differences were found in an analysis of gender and genotype by a case-only Pearson’s chi square test [rs1801275 *d*f(1) *P* = 0.31; rs1805015 *d*f(1) *P* = 0.29; rs1805011 *d*f(1) *P* = 0.56].

Stratification by site of CRC (colon versus rectum) was also carried out (data not shown). There was a statistically significant relationship between SNP rs1801275 (Q576R) and colon cancer risk, but not for rectal cancer. Carriage of at least one G allele conferred an OR for colon cancer of 1.27 (95% CI 1.02–1.58, *P* = 0.03, adjusted for sex and NSAID use) when compared with common AA homozygotes. The corresponding adjusted OR for rectal cancer was 1.09 (95% CI 0.84–1.42, *P* = 0.50).

For the survival analysis, some cases from the susceptibility study were excluded due to missing data or blood being drawn >2 years after CRC diagnosis. Therefore, 1145 cases were included in the survival analysis. There was a mean follow-up duration of 61.1 months (range 5.0–237.6 months). The 5 year survival from CRC-related death for all patients was 64.8%. As expected, survival differed according to Dukes’ stage at the time of diagnosis (Supplementary Table 2, available at *Carcinogenesis *Online; *P* < 0.001).

Supplementary Table 2, available at *Carcinogenesis *Online, shows the unadjusted hazard ratios and 95% CIs for the relationship between the IL-4Rα SNPs and CRC-specific mortality, as well as all-cause mortality. As expected, age (*P* < 0.001) and sex (*P* = 0.016) affected all-cause mortality. Histological differentiation and pathological stage of the primary CRC affected CRC-specific mortality (*P*_trend_ < 0.001), with a stronger effect on CRC survival. None of the six IL-4Rα SNPs showed significant effects on CRC or overall survival in unadjusted analyses or in analyses adjusting for age, sex, ever smoked, pathological stage and histological differentiation (data not shown). No significant relationship between the IL-4Rα SNPs and mortality were observed when the data were stratified by sex or tumour site (data not shown).

## Discussion

We have defined stage-specific effects of reduced IL-4Rα-mediated signalling on colorectal carcinogenesis in an established rodent model of chemical ‘sporadic’ colorectal carcinogenesis and confirmed the relevance of the pre-clinical observations in a case–control human epidemiological study of IL-4Rα SNPs.

We observed a clear distinction between increased tumour initiation (increased ACF multiplicity) and no effect on progression to adenoma and reduced tumour growth (demonstrated by no difference in AOM-induced tumour incidence and reduced tumour size) in *IL-4Rα*-null mice compared with WT animals. The association of rs1801275 (Q576R), a SNP affecting downstream IL-4Rα-mediated signalling linked to possible reduction in STAT-6 phosphorylation (22), with increased colonic cancer risk, but not CRC-related mortality, in females in our epidemiological study concurs with the stage-specific effects of *IL-4Rα* deletion demonstrated in the pre-clinical model. Therefore, IL-4Rα, like transforming growth factor β and forkhead box O3 for example (26,27), is a gene that appears to play different roles at different stages during colorectal carcinogenesis.

These data argue that pharmacological inhibition of IL-4Rα signalling, currently being developed for treatment of asthma and inflammatory bowel disease (28,29), could have deleterious consequences for future ‘sporadic’ CRC risk and any therapy involving IL-4R antagonism requires careful long-term evaluation in order to monitor colorectal adenoma and CRC risk. On the other hand, our pre-clinical data demonstrating smaller tumour size on an *IL-4Rα* ^*−/−*^ background hint that IL-4Rα antagonism could have a therapeutic role in treatment of established CRC. It would be very interesting to investigate the effect of treatment with the IL-4 mutein pitrakinra (28) on human CRC cell tumour xenograft growth in nude mice, in the first instance.

An interesting observation arising from our use of two different AOM administration protocols is that the number and size of ACFs did not change over time. It remains unclear whether ACFs are true precursor lesions, a small proportion of which can lead directly to macroscopic tumour formation, or whether ACFs are solely a surrogate mucosal biomarker of mucosal instability that predicts *de novo* tumour development elsewhere in the colon. Shpitz *et al.* (30) have provided indirect evidence that a small number of ACFs probably are true precursor lesions, but that the majority of ACFs are stable over time. Stable ACF multiplicity and size over time after AOM administration in our study are compatible with the concept that the ACF is, in fact, a biomarker of tumour growth. We are not aware of any previous literature comparing ACF multiplicity in early and late phases after AOM administration. However, the ACF number we report is consistent with existing literature on AOM-induced carcinogenesis in *BALB/c* mice (31).

The mechanism(s) by which reduced IL-4Rα-mediated signalling leads to reduced tumour growth remains unclear. We ruled out a simple effect on the balance between tumour cell proliferation and apoptosis. In fact, counter-intuitively, smaller *IL-4Rα* ‘knockout’ tumours exhibited reduced numbers of apoptotic cells compared with WT adenomas. The Fingleton laboratory has previously noted a difference in proliferation and apoptosis indices of *IL-4Rα* *-*null versus WT tumours depending on whether tumour growth occurred in a WT or *IL-4Rα* *-*null host (11). Lack of tumour cell IL-4Rα signalling alone in a WT host resulted in reduced tumour cell proliferation but no change in AI (11). However, combined lack of host and tumour IL-4Rα signalling was associated with an increase in AI, as well as a reduction in proliferation (11). The discrepancy between our data and those of Koller *et al.* (11) may be related to the use of dextran sulphate sodium in the latter study in order to model CAC rather than ‘sporadic’ colorectal carcinogenesis.

We did demonstrate that a lack of IL-4Rα-mediated signalling is associated with less nuclear and cytoplasmic β-catenin staining in epithelial cells in colonic adenomas. Similar findings have been reported by Koller *et al.* (11). WNT/β-catenin pathway dysregulation is critical for tumour progression and growth (32) suggesting that there may be a causal link between reduced tumour growth and β-catenin localization in *IL-4Rα* ^*−/−*^ tumours. Moreover, IL-4 drives catenin-related transcription in mouse thymocytes via GATA-3 and SATB1 transcription factors (33). Further work should now explore cross-talk between IL-4Rα- and catenin-related signalling in colorectal epithelial cells with either ‘loss of function’ adenomatous polyposis coli mutations or ‘gain of function’ *CTNNB1* (*β* *-catenin*) mutations.

Using a transgenic approach, we explored further the mechanism underlying the pro-tumorigenic effect of the lack of IL-4Rα signalling. The fact that *IL-13* deletion mirrored the effect of *IL-4Rα* deletion on AOM-induced ACF formation and that *IL-13* deletion did not abrogate the effect of lack of IL-4Rα-mediated signalling in DKO mice implicates a tumour suppressor role for the type II IL-4R (of which IL-4 and IL-13 are both ligands), rather than a tumour suppressor role of the type I IL-4R, or pro-tumorigenic activity of the IL-13Rα2 receptor. Several studies have previously reported localization of IL-4Rα chain protein to epithelial cells in 60–90% of human CRCs and in mouse CRC (11,34–36). Although upregulation of IL-4Rα chain protein expression in CRC compared with non-neoplastic colorectal epithelial cells is a consistent finding, there are conflicting data regarding whether IL-4Rα chain protein is actually expressed by non-neoplastic colorectal epithelial cells in humans (11,34–36).

We noted that *IL-4Rα* ^*−/−*^ mice failed to generate a CD11b^+^ Gr1^+^ MDSC response to colorectal tumours, consistent with the existing literature (16). Therefore, one hypothesis that requires testing is that reduced AOM-induced tumour growth, after the initiation phase, in *IL-4Rα* ^*−/−*^ mice is due to increased host antitumour immunocompetence resulting from an impaired IL-4Rα-dependent tumour-driven MDSC response. However, the small size of AOM-induced colorectal tumours did not allow us to investigate whether *IL-4Rα* ^*−/−*^ tumours contained less MDSCs and CD8^+^ lymphocytes by flow cytometry in keeping with this hypothesis. Recent reports describing the association of circulating and tumour-infiltrating MDSCs with CRC progression in clinical studies highlight the potential relevance of these cells to the functional consequences of reduced IL-4R function during colorectal carcinogenesis (37,38).

Our epidemiological findings that there is increased risk of CRC with each addition of the rs1801275 G allele contradict results published by Lee* et al.* (39). In their Korean case–control study, Lee *et al.* (39) reported decreased CRC risk associated with the 1902T>C SNP (rs1801275) (39). However, their study sample size was significantly smaller than our study (170 cases and 130 controls) with a consequent high risk of false-positive associations. In a slightly larger study than that of Lee *et al.* (39) (377 cases and 326 controls), Landi *et al.* (40) demonstrated no association between the same IL-4Rα SNP and CRC risk. Within our study, we have 72% power to detect a minimal OR of 1.8 for a rare homozygote of 4% frequency in the control population. Further work now needs to be conducted on independent data sets to confirm the association between IL-4Rα SNPs and CRC risk and survival.

Previous studies in haematopoietic cells have failed to conclude the functional consequences of the Q576R substitution on downstream signal transduction (19–22). However, the rs1801275 SNP has been associated with other pathologies including end-stage renal disease (41) and glioma (42) suggesting that Q576R may have functional consequences in non-haematopoietic cells. In the future, it will be essential to determine the effects of IL-4Rα SNPs including Q576R on human intestinal epithelial cell biology and kinase/phosphotyrosine phosphatase signal transduction. Interestingly, increased insulin receptor-like substrate 1 phosphorylation, which is a downstream consequence of the Q576R substitution in peripheral blood mononuclear cells (21), has recently been implicated in CRC progression (43).

## Conclusion

In summary, we provide pre-clinical evidence, backed up by human epidemiological observations, which together suggest that reduced type II IL-4R signalling is associated with increased ‘sporadic’ CRC risk, but no strong effect on CRC progression or clinical outcomes. These data should drive studies of the effect of pharmacological manipulation of type II IL-4R signalling in animal models of different stages of colorectal carcinogenesis with a view to determining the risk/benefit balance of targeting the IL-4R in different patient populations at risk of, or with established, CRC.

## Supplementary material

Supplementary Methods, Tables 1 and 2 and Figures 1–3 can be found at http://carcin.oxfordjournals.org/

## Funding

Yorkshire Cancer Research (L332) and Cancer Research UK (C588/A10589 and C37059/A11941). Engineering and Physical Sciences Research Council (EP/I000623/1 to N.I.). Medical Research Council (UK) Senior Clinical Fellowship (to M.A.H.).

*Conflict of Interest Statement:* None declared.

## Supplementary Material

Supplementary Data

## Abbreviations:

ACF: aberrant crypt focus
AI: apoptosis index
AOM: azoxy methane
BMI: body mass index
BrdU: 5-bromo-deoxyuridine
CAC: colitis-associated CRC
CI: confidence interval
CRC: colorectal cancer
DKO: double-knockout
IL-4R: interleukin-4 receptor
IQR: interquartile range
MDSC: myeloid-derived suppressor cells
NSAID: non-steroidal anti-inflammatory drug
OR: odds ratio
SD: standard deviation
SNP: single nucleotide polymorphism
STAT-6: signal transducer and activator of transcription-6
WT: wild-type.
