# Supplementary material for: Reduced type II interleukin-4 receptor signalling drives initiation, but not progression, of colorectal carcinogenesis: evidence from transgenic mouse models and human case–control epidemiological observations
Source: Carcinogenesis. 2013 Jun 19;34(10):2341–9. doi: 10.1093/carcin/bgt222 (PMC3786383; doi:10.1093/carcin/bgt222)
Supplement: Supplementary Data [file supp_34_10_2341__index.html]

Reduced type II interleukin-4 receptor signaling drives initiation, but not progression, of colorectal carcinogenesis: Evidence from transgenic mouse models and human case-control epidemiological observations — Reduced type II interleukin-4 receptor signaling drives initiation, but not progression, of colorectal carcinogenesis: Evidence from transgenic mouse models and human case-control epidemiological observations — Reduced type II interleukin-4 receptor signalling drives initiation, but not progression, of colorectal carcinogenesis: evidence from transgenic mouse models and human case–control epidemiological observations — Reduced type II interleukin-4 receptor signalling drives initiation, but not progression, of colorectal carcinogenesis: evidence from transgenic mouse models and human case–control epidemiological observations — Supplementary Data 

# Reduced type II interleukin-4 receptor signalling drives initiation, but not progression, of colorectal carcinogenesis: evidence from transgenic mouse models and human case–control epidemiological observations

## 

Data files

**Files in this Data Supplement:**

- Supplementary Data - Supplementary Data
- Supplementary Data - Supplementary Data
- Supplementary Data - Supplementary Data
- Supplementary Data - Supplementary Data
- Supplementary Data - Supplementary Data
- Supplementary Data - Supplementary Data
