# Supplementary material for: Reduced type II interleukin-4 receptor signalling drives initiation, but not progression, of colorectal carcinogenesis: evidence from transgenic mouse models and human case–control epidemiological observations
Source: Carcinogenesis. 2013 Jun 19;34(10):2341–9. doi: 10.1093/carcin/bgt222 (PMC3786383; doi:10.1093/carcin/bgt222)
Supplement: Supplementary Data [file supp_bgt222_IL_4Ra_Ingram_paper_Supplementary_Figure_3_Carcinogenesis.pptx]

## Slide 1
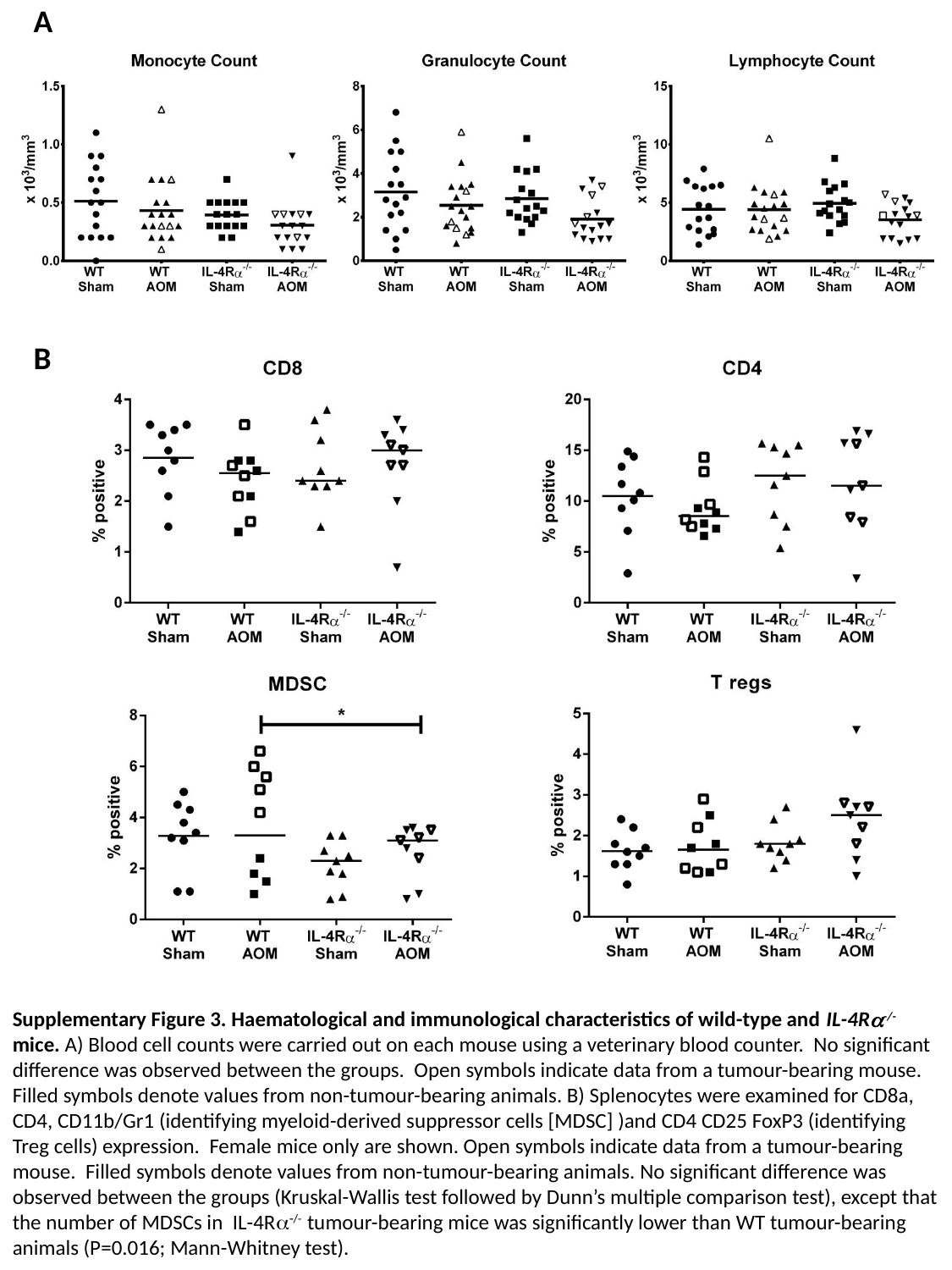

A
B
Supplementary Figure 3. Haematological and immunological characteristics of wild-type and IL-4Ra-/- mice. A) Blood cell counts were carried out on each mouse using a veterinary blood counter. No significant difference was observed between the groups. Open symbols indicate data from a tumour-bearing mouse. Filled symbols denote values from non-tumour-bearing animals. B) Splenocytes were examined for CD8a, CD4, CD11b/Gr1 (identifying myeloid-derived suppressor cells [MDSC] )and CD4 CD25 FoxP3 (identifying Treg cells) expression. Female mice only are shown. Open symbols indicate data from a tumour-bearing mouse. Filled symbols denote values from non-tumour-bearing animals. No significant difference was observed between the groups (Kruskal-Wallis test followed by Dunn’s multiple comparison test), except that the number of MDSCs in IL-4Ra-/- tumour-bearing mice was significantly lower than WT tumour-bearing animals (P=0.016; Mann-Whitney test).
