# Supplementary material for: Reduced type II interleukin-4 receptor signalling drives initiation, but not progression, of colorectal carcinogenesis: evidence from transgenic mouse models and human case–control epidemiological observations
Source: Carcinogenesis. 2013 Jun 19;34(10):2341–9. doi: 10.1093/carcin/bgt222 (PMC3786383; doi:10.1093/carcin/bgt222)
Supplement: Supplementary Data [file supp_bgt222_IL_4Ra_Ingram_paper_Supplementary_Table_2.doc]

**Supplementary Table 2. IL-4Rα** SNPs and CRC-specific and all-cause mortality

|  | **All-cause mortality** | | | | | **CRC-specific mortality*^a^*** | | | | |
| --- | --- | --- | --- | --- | --- | --- | --- | --- | --- | --- |
| **Characteristic*^b^*** | **Survived /censored** | **All cause mortality** | **HR*^c^*** | **95% CI*^c^*** | **p** | **Survived /censored** | **CRC-mortality** | **HR** | **95% CI** | **p** |
| **n** | **778** | **367** |  |  |  | **895** | **247** |  |  |  |
| **Age (mean)** | 66.5 | 69.7 | 1.03 | 1.02 - 1.04 | <0.001 | 67.3 | 67.8 | 1.01 | (1.00 - 1.02) | 0.17 |
| **Sex** |  |  |  |  |  |  |  |  |  |  |
| Male | 447 (57.5) | 235 (64.0) |  |  |  | 520 (58.1) | 160 (64.8) |  |  |  |
| Female | 331 (42.5) | 132 (36.0) | 0.77 | 0.62 - 0.95 | 0.016 | 375 (41.9) | 87 (35.2) | 0.74 | (0.57 - 0.97) | 0.027 |
| **Primary site** |  |  |  |  |  |  |  |  |  |  |
| Colon | 508 (65.3) | 242 (65.9) |  |  |  | 590 (65.9) | 159 (64.4) |  |  |  |
| Rectum | 270 (34.7) | 125 (34.1) | 0.97 | 0.78 - 1.20 | 0.76 | 305 (34.1) | 88 (35.6) | 1.05 | (0.81 - 1.36) | 0.72 |
| **Histological differentiation** |  |  |  |  |  |  |  |  |  |  |
| Well | 58 (8.2) | 21 (6.2) |  |  |  | 69 (8.4) | 10 (4.4) |  |  |  |
| Moderate | 561 (79.3) | 258 (75.9) | 1.56 | 1.00 - 2.44 | 0.050 | 648 (79.1) | 168 (74.7) | 1.92 | (1.01 - 3.64) | 0.045 |
| Poor | 88 (12.4) | 61 (17.9) | 2.32 | 1.41 - 3.81 | 0.001 | 102 (12.5) | 47 (20.9) | 3.26 | (1.64 - 6.44) | 0.001 |
| **trend** |  |  | 1.51 | 1.21 - 1.88 | <0.001 |  |  | 1.74 | (1.33 - 2.86) | <0.001 |
| **Pathological stage** |  |  |  |  |  |  |  |  |  |  |
| A | 173 (22.9) | 32 (8.9) |  |  |  | 195 (22.4) | 10 (4.1) |  |  |  |
| B | 292 (38.6) | 110 (30.7) | 1.70 | 1.14 - 2.51 | 0.009 | 354 (40.6) | 47 (19.5) | 2.37 | (1.20 - 4.70) | 0.013 |
| C | 259 (34.2) | 133 (37.2) | 2.28 | 1.55 - 3.35 | 0.000 | 289 (33.2) | 101 (41.9) | 5.61 | (2.93 - 10.7) | 0.000 |
| D | 33 (4.4) | 83 (23.2) | 12.8 | 8.47 - 19.4 | 0.000 | 33 (3.8) | 83 (34.4) | 33.4 | (17.3 - 64.7) | 0.000 |
| **trend** |  |  | 2.19 | 1.91 - 2.51 | <0.001 |  |  | 3.62 | (3.03 – 4.33) | <0.001 |
| **Ever smoked** |  |  |  |  |  |  |  |  |  |  |
| No | 308 (40.0) | 107 (33.4) |  |  |  | 340 (39.2) | 74 (33.6) |  |  |  |
| Yes | 462 (60.0) | 213 (66.6) | 1.25 | 0.99 - 1.57 | 0.063 | 527 (60.8) | 146 (66.4) | 1.22 | (0.92 - 1.61) | 0.17 |
| **NSAID use** |  |  |  |  |  |  |  |  |  |  |
| No | 548 (71.2) | 228 (71.7) |  |  |  | 610 (70.4) | 163 (74.4) |  |  |  |
| Yes | 222 (28.8) | 90 (28.3) | 0.95 | 0.74 - 1.21 | 0.66 | 256 (29.6) | 56 (25.6) | 0.84 | (0.62 - 1.14) | 0.27 |
| **rs1801275** |  |  |  |  |  |  |  |  |  |  |
| AA | 439 (57.9) | 216 (59.8) |  |  |  | 505 (57.8) | 148 (60.9) |  |  |  |
| AG | 276 (36.4) | 126 (34.9) | 0.89 | 0.71 - 1.11 | 0.301 | 319 (36.5) | 82 (33.7) | 0.86 | (0.66 - 1.13) | 0.288 |
| GG | 43 (5.7) | 19 (5.3) | 0.90 | 0.56 - 1.43 | 0.651 | 49 (5.6) | 13 (5.3) | 0.90 | (0.51 - 1.60) | 0.730 |
| **trend** |  |  | 0.92 | 0.77 - 1.09 | 0.325 |  |  | 0.90 | (0.73 - 1.12) | 0.348 |
| **rs1805010** |  |  |  |  |  |  |  |  |  |  |
| AA | 233 (31.4) | 101 (29.3) |  |  |  | 256 (30.0) | 76 (32.6) |  |  |  |
| AG | 369 (49.7) | 173 (50.1) | 1.10 | 0.86 - 1.41 | 0.433 | 428 (50.2) | 114 (48.9) | 0.93 | (0.70 - 1.25) | 0.631 |
| GG | 141 (19.0) | 71 (20.6) | 1.06 | 0.78 - 1.43 | 0.722 | 168 (19.7) | 43 (18.5) | 0.84 | (0.58 - 1.22) | 0.365 |
| **trend** |  |  | 1.03 | 0.89 - 1.20 | 0.657 |  |  | 0.92 | (0.77 - 1.10) | 0.366 |
| **rs1805011** |  |  |  |  |  |  |  |  |  |  |
| AA | 555 (76.0) | 272 (77.1) |  |  |  | 644 (76.3) | 181 (76.7) |  |  |  |
| AC/CC | 175 (24.0) | 81 (22.9) | 0.96 | 0.75 - 1.22 | 0.719 | 200 (23.7) | 55 (23.3) | 0.99 | (0.73 - 1.34) | 0.943 |
| **rs1805013** |  |  |  |  |  |  |  |  |  |  |
| CC | 690 (90.6) | 321 (90.2) | 1.0 |  |  | 789 (90.0) | 219 (92.0) |  |  |  |
| CT/TT | 72 (9.4) | 35 (9.8) | 1.00 | 0.70 - 1.41 | 0.980 | 88 (10.0) | 19 (8.0) | 0.76 | (0.48 - 1.21) | 0.250 |
| **rs1805015** |  |  |  |  |  |  |  |  |  |  |
| TT | 500 (65.5) | 238 (66.9) |  |  |  | 575 (65.5) | 161 (67.6) |  |  |  |
| TC | 233 (30.5) | 105 (29.5) | 0.95 | 0.76 - 1.20 | 0.675 | 269 (30.6) | 68 (28.6) | 0.91 | (0.68 - 1.21) | 0.507 |
| CC | 30 (3.9) | 13 (3.7) | 0.91 | 0.52 - 1.59 | 0.747 | 34 (3.9) | 9 (3.8) | 0.95 | (0.49 - 1.86) | 0.884 |
| **trend** |  |  | 0.95 | 0.79 - 1.15 | 0.618 |  |  | 0.93 | (0.74 - 1.18) | 0.564 |
| **rs1805016** |  |  |  |  |  |  |  |  |  |  |
| TT | 684 (89.2) | 313 (88.2) |  |  |  | 780 (88.4) | 214 (90.3) |  |  |  |
| TG/GG | 83 (10.8) | 42 (11.8) | 1.06 | 0.77 - 1.47 | 0.709 | 102 (11.6) | 23 (9.7) | 0.82 | (0.53 - 1.26) | 0.368 |

*^a^*3 individuals were dropped from the CRC mortality analysis due to unknown cause of death; *^b^*sample number and percentage are shown for all variables except age (mean); *^c^*HR, hazard ratio; CI, confidence interval
