# Supplementary material for: Reduced type II interleukin-4 receptor signalling drives initiation, but not progression, of colorectal carcinogenesis: evidence from transgenic mouse models and human case–control epidemiological observations
Source: Carcinogenesis. 2013 Jun 19;34(10):2341–9. doi: 10.1093/carcin/bgt222 (PMC3786383; doi:10.1093/carcin/bgt222)
Supplement: Supplementary Data [file supp_bgt222_IL_4Ra_Ingram_paper_Supplementary_Figure_2_Carcinogenesis.pptx]

## Slide 1
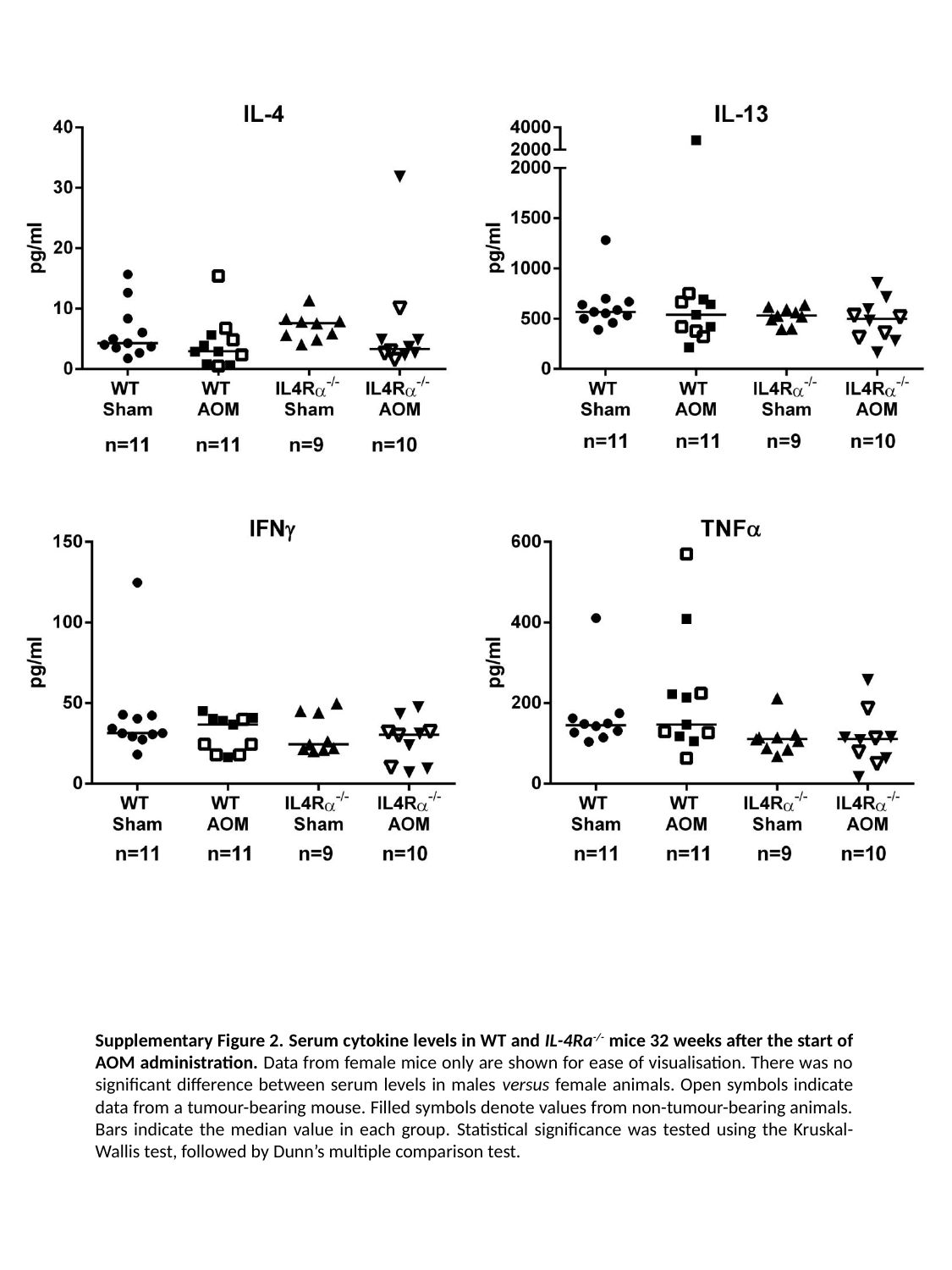

Supplementary Figure 2. Serum cytokine levels in WT and IL-4Ra-/- mice 32 weeks after the start of AOM administration. Data from female mice only are shown for ease of visualisation. There was no significant difference between serum levels in males versus female animals. Open symbols indicate data from a tumour-bearing mouse. Filled symbols denote values from non-tumour-bearing animals. Bars indicate the median value in each group. Statistical significance was tested using the Kruskal-Wallis test, followed by Dunn’s multiple comparison test.
