# Supplementary material for: Reduced type II interleukin-4 receptor signalling drives initiation, but not progression, of colorectal carcinogenesis: evidence from transgenic mouse models and human case–control epidemiological observations
Source: Carcinogenesis. 2013 Jun 19;34(10):2341–9. doi: 10.1093/carcin/bgt222 (PMC3786383; doi:10.1093/carcin/bgt222)
Supplement: Supplementary Data [file supp_bgt222_IL_4Ra_Ingram_paper_Supplementary_methods.doc]

**Supplementary Methods**

**Animal studies**

All procedures were carried out in accordance with the Home Office Animal Scientific Procedures Act 1986. All mice were weighed weekly as part of ongoing general health checks. All blood and tissues were harvested and analysed blind to genotype and treatment allocation.

**PCR genotype analysis**

Genomic DNA was isolated from ear notches using a GenElute Kit (Sigma).

IL-4Rα ^-/-^ mice were genotyped using separate PCR reactions for wild-type and ‘knock-out’ alleles (Ko *et al*, *J Pathol* 2008;**214**:603-9). 0.5 μl of each of the following primers (100 μM) was added in a reaction containing 1 mM Mg^2+^:

Wild-type forward primer 5’ TGA CCT ACA AGG AAC CCA GGC 3’

Wild-type reverse primer 5’ CTC GGC GCA CTG ACC CAT CT 3’

‘Knock-out’ forward primer 5’ GGC TGC TGA CCT GGA ATA ACC 3’

‘Knock-out’ reverse primer 5’ CCT TTG AGA ACT GCG GGC T 3’

Thermal cycler conditions were: 94^o^C for 2 minutes, then 40 cycles of 94^o^C for 30 seconds, 57^o^C for 30 seconds and 72^o^C for 1 minute with a final extension of 72^o^C for 5 minutes. Wild-type mice generated a DNA product of 612 base pairs (bp) and the null allele generated a DNA product of 471 bp.

*IL-13 ^-/-^* mice were genotyped using 0.5 μl of each of the following four primers (10 OD/ml) in a reaction containing 2.5 mM Mg^2+^:

Primer 1 5’ GGG TGA CTG CAG TCC TGG CT 3’

Primer 2 5’ GTT GCT CAG CTC CTC AAT AAG C 3’

Primer 3 5’ GGC GGA TGA GCG GCA TTT TCC GTG 3’

Primer 4 5’ GCC GAA AGG CGC GGT GCC GCT GGC 3’

Thermal cycler conditions were: 94^o^C for 2 minutes, then 30 cycles of 94^o^C for 30 seconds, 64^o^C for 30 seconds and 72^o^C for 30 seconds with a final extension of 72^o^C for 10 minutes. Mice with wild-type *IL-13* alleles generated a DNA product of 100 bp whereas *IL-13 ^-/-^* mice generated a DNA product of 200 bp.

**Blood collection and analysis**

Five drops of blood were placed in a 1.3 mL paediatric K_3_ EDTA blood tube (Greiner Bio-One Limited, Gloucester, United Kingdom). Whole blood was analysed 5 minutes later using a haemocytometer (Scil Vet animal blood counter, Viernheim, Germany) calibrated for mouse blood. The remainder of the blood was allowed to clot on ice and was then centrifuged at 8000 *g* for 10 minutes. Serum was aliquoted and stored at -80^o^C before immunoassay.

**Cytokine analysis**

Levels of 8 cytokines (IL-1β, IL-4, IL-13, IL-6, γ interferon [IFN], tumour necrosis factor [TNF]α, IL-15 and IL-10) in serum were measured using a customised multiplex immunoassay as per manufacturer’s instructions (Bio-Rad Laboratories, Hemel Hempstead, UK). Serum was diluted 1 in 4 with PBS prior to assay. Assay plates were read using a Luminex-100 plate-reader (Luminex Corporation, Texas, U.S.A.) and Bio-Plex Manager software version 4.1 (Bio-Rad Laboratories).

**Flow cytometry**

Dead cells were excluded by 7-amino actinomyosin D uptake. For detection of regulatory T cells, splenocytes were first incubated with fluorescein isothiocyanate (FITC)-conjugated anti-CD4 antibody and APC-conjugated anti-CD25 antibody before fixation and permeabilisation using a Mouse Regulatory T cell Staining Kit (eBioscience, San Diego, CA, USA). The cells were then incubated with a PE-conjugated anti-Foxp3 antibody before flow cytometry.

**Antibodies used for flow cytometry**

| **Antibody (clone)** | **Supplier** | **Conjugate*^a^*** | **μl antibody per 10^6^ cells in 100μl volume (μg antibody)** |
| --- | --- | --- | --- |
| CD16/32 (2.4G2) | BD Biosciences | None | 1 (0.5) |
| CD4 (RM4-5) | BD Biosciences | AlexaFluor® 700 | 1.25 (0.25) |
| IgG_2a_ (R35-95) | BD Biosciences | AlexaFluor® 700 | 1.25 (0.25) |
| CD8a (53-6.7) | BD Biosciences | Pacific Blue™ | 2.5 (0.5) |
| IgG_2a_ (R35-95) | BD Biosciences | Pacific Blue™ | 2.5 (0.5) |
| Gr1 (RB6-8C5) | BD Biosciences | FITC | 2 (1) |
| IgG_2b_ (A95-1) | BD Biosciences | FITC | 2 (1) |
| CD11b (M1/70) | BD Biosciences | PE | 5 (1) |
| IgG_2b_ (A95-1) | BD Biosciences | PE | 5 (1) |
| CD4 (RM4-5) | eBiosciences | FITC | 0.25 (0.125) |
| IgG_2a_ | eBiosciences | FITC | 10 (0.125) |
| CD25 (PC61.5) | eBiosciences | APC | 0.3 (0.06) |
| IgG_1_ | eBiosciences | APC | 0.3 (0.06) |
| Foxp3 (FJK-16s) | eBiosciences | PE | 2.5 (0.5) |
| IgG_2a_ | eBiosciences | PE | 2.5 (0.5) |

*^a^*FITC, Fluorescein isothiocyanate; PE, R-Phycoerythrin; APC, Allophycocyanin

**Immunohistochemistry**

Four μm-thick fixed sections of adenomas were de-waxed and rehydrated through a xylene and ethanol series. Endogenous peroxidase was blocked using 0.3% (v/v) H_2_O_2_ in methanol. For β-catenin and cleaved caspase 3 staining, slides were subject to microwave oven antigen retrieval in 0.01 M citrate buffer, pH 6 for 10 minutes. Antibody diluent solution (Zymed Laboratories, San Francisco, USA) was briefly applied to each section to block non-specific binding. β-catenin or cleaved caspase 3 antibody (Cell Signaling Technology, Inc, Denvers, MA, USA) was then applied to the section at 1:200 or 1:250 dilution in antibody diluent solution (Zymed Laboratories) respectively for one hour at room temperature and a rabbit Envision+™ kit (DAKOcytomation, Ely, UK) was used to localize immunoreactivity.

For BrdU staining, slides were incubated with casein in PBS (Vector Laboratories) for 20 minutes. Anti-BrdU antibody was diluted in a nuclease solution following manufacturer’s instructions (Cell Proliferation Detection Kit, GE Healthcare). This was applied to the sections for 10 minutes before washing and application of mouse Envision+™ kit reagent (DAKOcytomation).

All sections were counterstained with haematoxylin, dehydrated in ethanol, cleared in xylene and mounted in DePex. Images were acquired using a Nikon Eclipse E1000 microscope with NIS Elements software (Nikon Instruments Europe, Amsterdam, The Netherlands).

#### Immunohistochemistry scoring

For BrdU and cleaved caspase 3 scoring, positive-stained and negative nuclei in dysplastic epithelial tumour cells were counted in 4 high-power fields per adenoma and reported as the percentage positivity of dysplastic epithelial cells by an observer blinded to the slide identity. BrdU positive cells were also counted in normal whole longitudinal colonic crypt sections and the data were expressed as the number of BrdU-positive cells per crypt.

For β-catenin scoring, 4 high-power (x600) fields from each section were photographed and every dysplastic epithelial cell was scored as either; no staining, membranous staining, cytoplasmic staining or nuclear staining by an observer blinded to the slide identity (Wong *et al*, *Mol Pathol* 2003;**56**:347-52). The percentage number of epithelial cells per tumour that had either or both nuclear and cytoplasmic staining was calculated and the mean value for all tumours of the same genotype was derived.

**Genetic epidemiology study (further methodology)**

Cases and controls provided a venous blood sample of at least 10 ml, which was stored in an EDTA blood tube at -20^o^C. Genomic DNA was extracted using a modified BACC2 extraction protocol (Gen-Probe Life Sciences, Manchester, UK).

**Risk analysis**

The genotypes for each SNP were firstly analysed as a three-group categorical variable and then a test for trend on the number of minor alleles was performed. These models were then repeated, stratifying firstly by sex and then by tumour site (colon/rectum), to assess differential effects. Heterozygotes were combined with rare homozygotes due to small numbers. Likelihood ratio tests were performed to detect significant SNP-sex interactions.

**Mortality analysis**

In these analyses, heterozygotes were grouped with rare homozygotes for SNPs rs1805016, rs1805013 and rs1805011 due to small numbers. These analyses were repeated adjusting for age, sex, ever smoked, histological tumour differentiation (well, moderate, poor) and pathological stage (Dukes’ stage A-D). The proportional hazards assumption was checked by testing for a non-zero slope in a generalized linear regression of the scaled Schoenfeld residuals on functions of time.
