# Supplementary material for: Reduced type II interleukin-4 receptor signalling drives initiation, but not progression, of colorectal carcinogenesis: evidence from transgenic mouse models and human case–control epidemiological observations
Source: Carcinogenesis. 2013 Jun 19;34(10):2341–9. doi: 10.1093/carcin/bgt222 (PMC3786383; doi:10.1093/carcin/bgt222)
Supplement: Supplementary Data [file supp_bgt222_IL_4Ra_Ingram_paper_Supplementary_Table_1.doc]

**Supplementary Table 1. Sex-stratified analyses of the association between IL-4Rα SNPs and CRC susceptibility**

|  |  | **Males** | | | | | | **Females** | | | | | | **LR test*^a^*** |
| --- | --- | --- | --- | --- | --- | --- | --- | --- | --- | --- | --- | --- | --- | --- |
| **SNP** |  | **Controls** | **Cases** | **Unadjusted OR**  **(95% CI)** | **p** | **Adjusted OR*^b^***  **(95% CI)** | **p** | **Controls** | **Cases** | **Unadjusted OR**  **(95% CI)** | **p** | **Adjusted OR*^b^***  **(95% CI)** | **p** | **p** |
|  |  | **n (%)** | **n (%)** |  |  |  |  | **n (%)** | **n (%)** |  |  |  |  |  |
| **Rs1801275**  **Q576R** | **AA** | 156 (57.8) | 506 (59.6) |  |  |  |  | 208 (67.8) | 341 (56.9) |  |  |  |  |  |
|  | **AG/GG** | 114 (42.2) | 343 (40.4) | 0.93 (0.70 - 1.22) | 0.60 | 0.93 (0.70 - 1.23) | 0.59 | 99 (32.2) | 258 (43.1) | **1.59 (1.19 - 2.12)** | **0.002** | **1.63 (1.21 - 2.19)** | **0.001** | **0.007** |
| **Rs1805015**  **S503P** | **TT** | 173 (64.6) | 566 (66.8) |  |  |  |  | 227 (73.7) | 385 (64.2) |  |  |  |  |  |
|  | **TC/CC** | 95 (35.4) | 281 (33.2) | 0.90 (0.68 - 1.21) | 0.49 | 0.89 (0.67 - 1.20) | 0.46 | 81 (26.3) | 215 (35.8) | **1.57 (1.16 - 2.12)** | **0.004** | **1.59 (1.17 - 2.17)** | **0.003** | **0.008** |
| **Rs1805016** | **TT** | 242 (90.6) | 764 (89.7) |  |  |  |  | 283 (91.6) | 524 (88.1) |  |  |  |  |  |
| **A752S** | **TG/GG** | 25 (9.4) | 88 (10.3) | 1.11 (0.70 - 1.78) | 0.65 | 1.14 (0.71 - 1.83) | 0.58 | 26 (8.4) | 71 (11.9) | 1.47 (0.92 - 2.36) | 0.11 | 1.50 (0.92 - 2.44) | 0.10 | 0.44 |
| **Rs1805013**  **S436L** | **CC** | 253 (93.0) | 774 (91.1) |  |  |  |  | 283 (91.6) | 534 (89.6) |  |  |  |  |  |
|  | **CT/TT** | 19 (7.0) | 76 (8.9) | 1.31 (0.78 - 2.20) | 0.31 | 1.33 (0.79 - 2.26) | 0.28 | 26 (8.4) | 62 (10.4) | 1.26 (0.78 - 2.04) | 0.34 | 1.33 (0.81 - 2.18) | 0.26 | 0.98 |
| **Rs1805011** | **AA** | 193 (72.3) | 629 (76.6) |  |  |  |  | 246 (81.7) | 444 (75.3) |  |  |  |  |  |
| **A400E** | **AC/CC** | 74 (27.7) | 192 (23.4) | 0.80 (0.58 - 1.09) | 0.15 | 0.77 (0.56 - 1.06) | 0.11 | 55 (18.3) | 146 (24.7) | **1.47 (1.04 - 2.08)** | **0.029** | **1.56 (1.09 - 2.22)** | **0.015** | **0.004** |
| **Rs1805010**  **I75V** | **AA** | 79 (29.9) | 255 (30.8) |  |  |  |  | 83 (27.8) | 173 (29.8) |  |  |  |  |  |
|  | **AG/GG** | 185 (70.1) | 572 (69.2) | 0.96 (0.71 - 1.30) | 0.78 | 0.95 (0.70 - 1.29) | 0.74 | 216 (72.2) | 408 (70.2) | 0.91 (0.67 - 1.23) | 0.53 | 0.91 (0.66 - 1.25) | 0.55 | 0.83 |

*^a^*LR test, likelihood ratio test; *^b^*adjusted for NSAID use
