# Supplementary material for: Reduced type II interleukin-4 receptor signalling drives initiation, but not progression, of colorectal carcinogenesis: evidence from transgenic mouse models and human case–control epidemiological observations
Source: Carcinogenesis. 2013 Jun 19;34(10):2341–9. doi: 10.1093/carcin/bgt222 (PMC3786383; doi:10.1093/carcin/bgt222)
Supplement: Supplementary Data [file supp_bgt222_IL_4Ra_Ingram_paper_Supplementary_Figure_1_Carcinogenesis.pptx]

## Slide 1
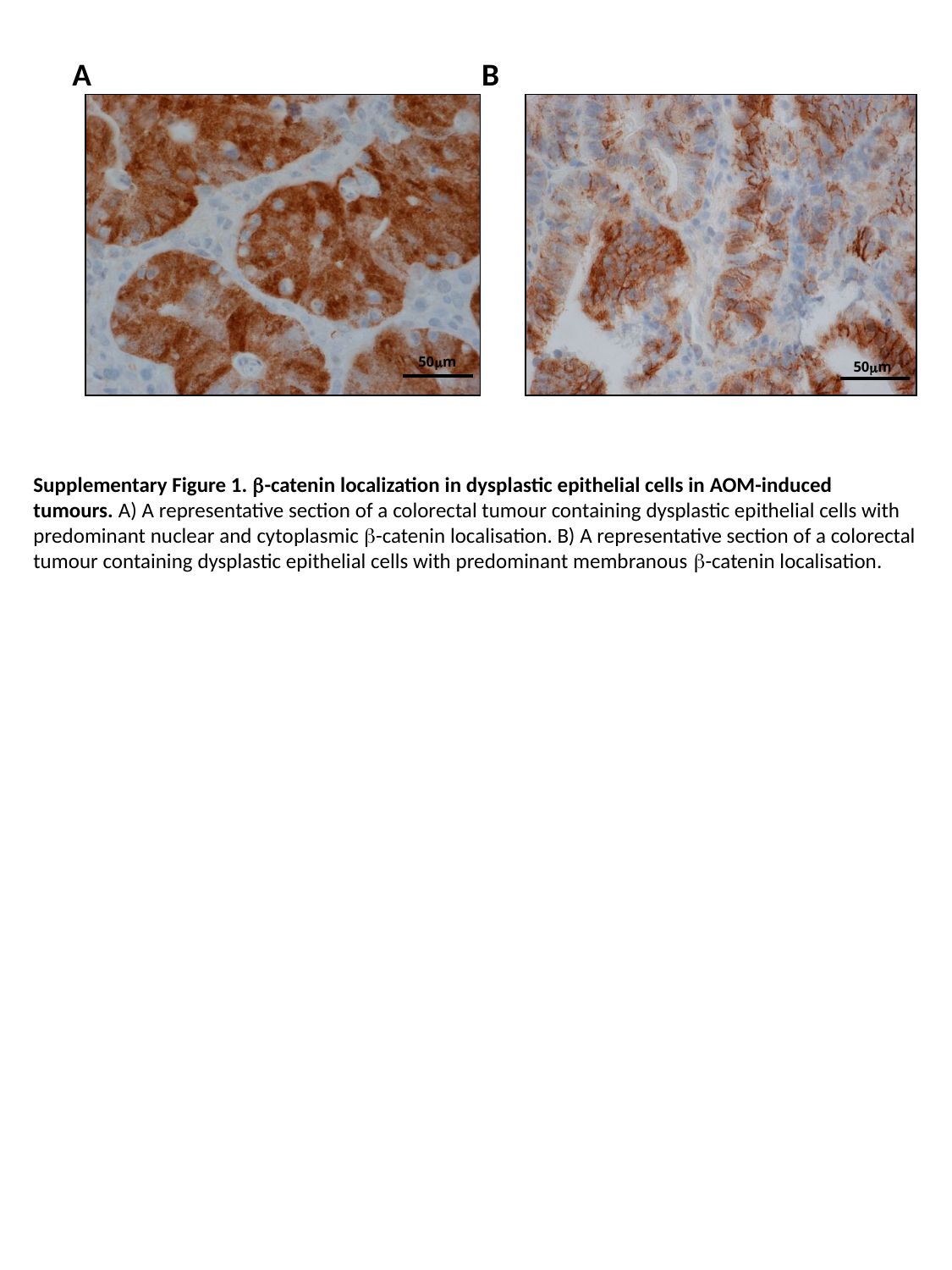

A
B
50mm
50mm
Supplementary Figure 1. b-catenin localization in dysplastic epithelial cells in AOM-induced tumours. A) A representative section of a colorectal tumour containing dysplastic epithelial cells with predominant nuclear and cytoplasmic b-catenin localisation. B) A representative section of a colorectal tumour containing dysplastic epithelial cells with predominant membranous b-catenin localisation.
